# Iron dyshomeostasis in neuropsychiatric disorders

**DOI:** 10.3389/fpsyt.2026.1771423

**Published:** 2026-04-21

**Authors:** Mengjie Cheng, Jiazi Ma, Yong Yang, Mao Cao, Enguo Zhang, Bin Feng, Qiang Wang, Zhongjun Du

**Affiliations:** 1 Shandong Academy of Occupational Health and Occupational Medicine, Shandong First Medical University and Shandong Academy of Medical Sciences, Jinan, Shandong, China; 2 Department of Preventive Medicine and Public Health Laboratory Sciences, School of Medicine, Jiangsu University, Zhenjiang, Jiangsu, China; 3 Department of Occupational Diseases, The Second Hospital of Harbin, Harbin, Heilongjiang, China

**Keywords:** Alzheimer’s disease, ferroptosis, iron metabolism, neurodegenerative diseases, neuropsychiatric disorders, Parkinson’s disease

## Abstract

Iron is an indispensable element for the normal physiological function of the brain. In terms of neuronal metabolism, iron is involved in multiple critical biological processes such as oxygen transport, energy metabolism, DNA synthesis, neurotransmitter synthesis and myelin formation. Maintaining brain iron homeostasis is crucial for neurodevelopment and function. Iron dyshomeostasis has been associated with the onset and progression of various neuropsychiatric disorders, including Parkinson’s disease, Alzheimer’s disease, depression, schizophrenia, attention deficit hyperactivity disorder, and autism spectrum disorder. In neurodegenerative diseases such as Parkinson’s disease and Alzheimer’s disease, abnormally elevated iron levels can be detected in specific brain regions, including the basal ganglia and the prefrontal cortex. These changes are often accompanied by pathological processes such as oxidative stress, neuroinflammation, and pathological protein aggregation. Therefore, brain iron metabolism is an important entry point for understanding the pathophysiological process of neuropsychiatric disorders. Mechanistically, iron overload induces oxidative damage through the Fenton reaction, exacerbating mitochondrial dysfunction and abnormal protein aggregation. The effects of iron deficiency vary across different diseases; its impact on myelination and neurotransmitter synthesis may increase the risk of neurodevelopmental disorders such as attention deficit hyperactivity disorder (ADHD), while its effects on immune activation and energy metabolism may contribute to the development of mental disorders such as depression. This article systematically reviews the current research progress of the role of cerebral iron metabolism in neuropsychiatric diseases. It focuses on the mechanisms underlying iron homeostasis imbalances in neurodegenerative and psychiatric diseases. Building on this foundation, the article analyzes the therapeutic targets and clinical significance of iron metabolism-related interventions and outlines future research directions in this field.

## 1 Introduction

Although the human brain accounts for only 2% of body weight, it consumes 20% of the body’s oxygen and energy (1). Behind this huge metabolic demand, iron, as a necessary trace element for the human body, plays a central role in the physiological activities of the brain (2). It not only participates in the transportation of oxygen by hemoglobin to ensure the oxygen supply to brain tissues, but also acts as a cofactor for various enzymes, directly participating in energy metabolism within mitochondria, DNA synthesis and repair, and the synthesis of key neurotransmitters such as dopamine and serotonin (3). Moreover, iron is indispensable for oligodendrocytes in synthesizing myelin, which is crucial for effective insulation of axons and rapid conduction of nerve impulses (4). Therefore, the precise regulation of iron homeostasis in the brain is the foundation for maintaining normal neural development, cognitive functions, and emotional regulation (5, 6). When iron homeostasis is disrupted, iron exhibits redox activity; excess free iron can catalyze the production of destructive reactive oxygen species through the Fenton reaction, thereby causing sustained oxidative damage to lipids, proteins, and DNA (7).

Recent research findings indicate that, although neurodegenerative diseases and various mental disorders present with distinct clinical manifestations, they all share a characteristic iron dyshomeostasis in the brain as part of their pathological processes. This disorder manifests in two distinct ways. Taking Parkinson’s disease as an example, it presents as abnormal iron deposition in specific brain regions, leading to oxidative stress, neuroinflammation, and mitochondrial dysfunction; whereas in patients with ADHD, it manifests as functional iron deficiency, which affects myelination and the function of neurotransmitter systems such as dopamine (8). Whether it is the significant accumulation of iron in the substantia nigra of Parkinson’s disease patients or the deficiency of iron in the striatum of individuals with ADHD, both indicate that iron metabolism imbalance is a critical factor in understanding the neuropathological changes underlying these conditions (9). Iron deficiency or excess can affect the function of brain tissue. Therefore, iron homeostasis of the brain must be precisely adjusted (10). A deep understanding of the precise regulation of iron homeostasis is crucial for exploring new treatment strategies for neuropsychiatric disorders.

## 2 Physiology and pathophysiology of iron metabolism in the brain

### 2.1 Iron uptake and metabolism

**Figure 1:**
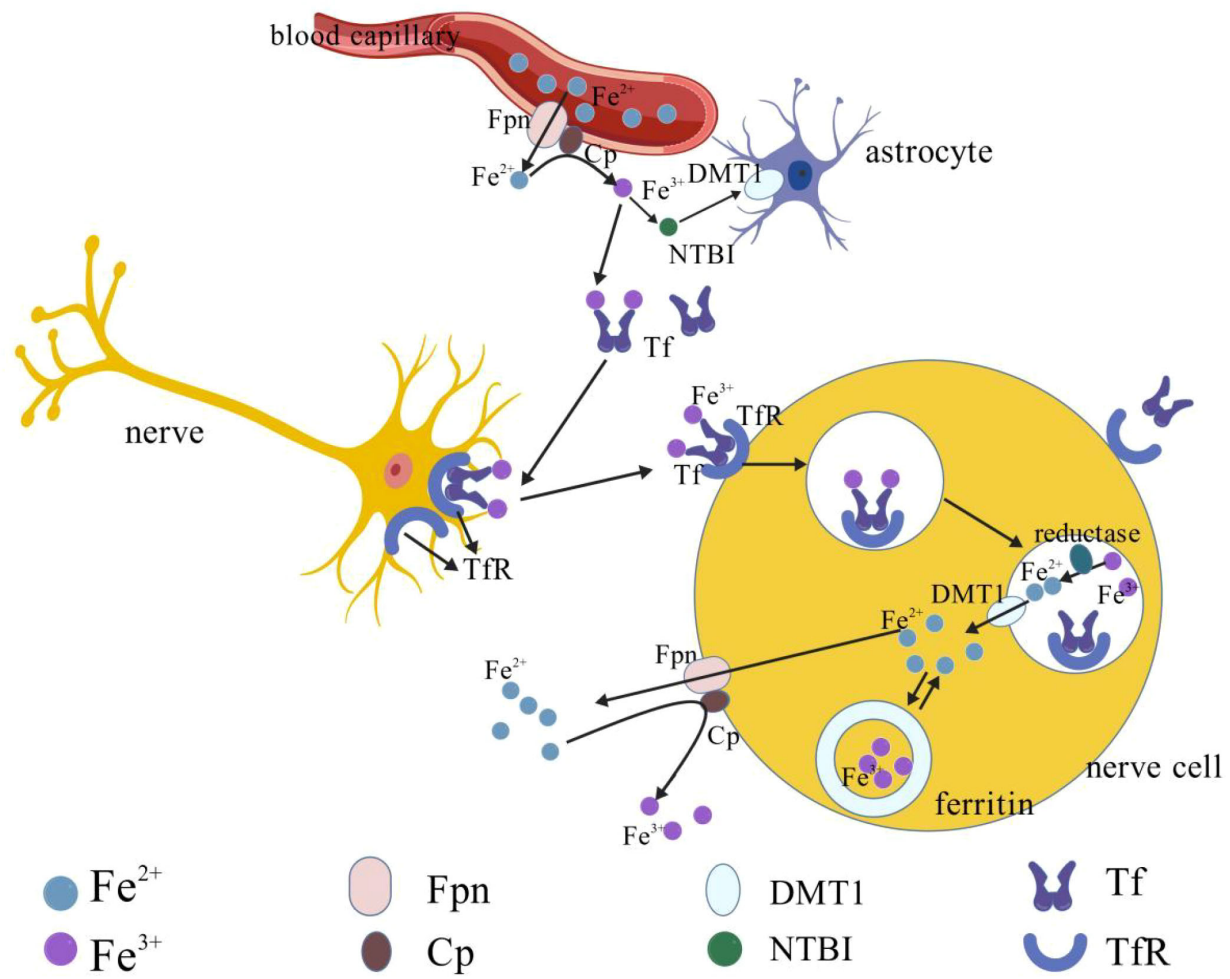
The process of brain iron uptake, transport, and metabolism. After Fe^2+^ is released from the blood vessels, it is oxidized into Fe^3+^ by ceruloplasmin on the foot process of astrocytes and binds to ferritin. The complex enters the cell by binding to the surface receptor of the nerve cell, and Fe^3+^ is reduced to Fe^2+^ in the vesicle and transferred to the cytoplasm through DMT1. Intracellular Fe^2+^ is partially involved in metabolism and partially stored in ferritin. Excess Fe^2+^ can be excreted through FPN. Astrocytes can directly ingest Fe^3+^ through DMT1. The discharged Fe^2+^ is oxidized to Fe^3+^ by ceruloplasmin to maintain the dynamic balance of iron in the brain.

Iron metabolism in the brain is a highly complex and finely regulated process. In the central nervous system, iron mainly enters the brain through the blood-brain barrier and the blood-cerebrospinal fluid barrier. Its transport is mainly mediated by the transferrin (Tf)-transferrin receptor (TfR) and divalent metal transporter 1 (DMT1)-ferroportin (FPN) pathways (11). The specific mechanism is as follows (12): (as shown in **Figure 1**). In the endothelial cells of the brain’s capillaries, endocytosis mediated by transferrin receptor 1 allows Fe^3+^ bound to Tf in the blood to enter the cells. In the acidic environment of the endocytic vesicles, Fe^3+^ is reduced to Fe^2+^, and Fe^2+^ is released into the cytoplasm through divalent metal ion transporter 1 (13). (as shown in the bottom right corner of **Figure 1**). Subsequently, Fe^2+^ is exported to the brain interstitial fluid by the basolateral membrane iron exporter, ferroportin 1 (FPN1). At this time, ceruloplasmin on the endothelial cell membrane rapidly oxidizes the released Fe^2+^ to Fe^3+^ for subsequent use (14). The Fe^3+^ entering the interstitial fluid combines with Tf to form a transferrin-iron complex (15). Neurons mainly take up iron through the highly expressed TfR via the endocytosis pathway. The transferrin-iron complex binds to the receptor and is internalized. In the acidic endosome, Fe^3+^ dissociates and is reduced to Fe^2+^, and is finally transported to the cytoplasm via DMT1 (16). The iron in the cytoplasm can be directly utilized or stored in ferritin in the form of Fe^3+^ (17). Astrocytes not only take up iron through TfR but can also directly take up iron ions from the interstitial fluid through proteins such as divalent metal ion transporter 1 (18). Moreover, astrocytes are the main producers of ceruloplasmin in the brain (19), which is crucial for maintaining the iron output function of Fpn and may participate in iron oxidation and transfer between cells (20). The excretion of iron in the brain mainly occurs in the choroid plexus. The iron in cerebrospinal fluid and interstitial fluid is taken up by the epithelial cells of the choroid plexus and is expelled to the blood through their basolateral membrane iron transporter, completing the iron cycle (21). (As shown in **Figure 1**). Multiple cell types and transport proteins collectively form a complex regulatory network that ensures the precise distribution and dynamic equilibrium of iron within the brain. Research data (22) shows that microglia also play an important role in iron storage and metabolism, as they express ferritin to store iron and release it for use by neurons when necessary.

The figure was drawn using the BioGDP General Biomedical Graphic Drawing Platform (https://biogdp.com/).

### 2.2 Regulation of iron homeostasis

Under normal circumstances, there is an iron steady state in the brain. The homeostasis of iron metabolism is precisely regulated by many genes, and any abnormal expression of related genes will lead to an imbalance of iron homeostasis in the body. The homeostasis of iron metabolism in the body is precisely regulated by the axis of Hepcidin Antimicrobial Peptide (HAMP)-FPN. The system dynamically regulates the absorption, release and distribution of iron by sensing changes in intracellular iron levels (23, 24). At the molecular level, HAMP is the general switch of the overall iron steady state in the body. When the body is in a state of iron overload, HAMP plays a very crucial inhibitory role. It binds to the FPN on the target cell membrane, internalizes and degrades FPN in lysosomes, thus inhibiting the absorption of iron by intestinal cells and the release of iron into the serum by macrophages or hepatocytes, regulating the uptake and distribution of iron (25). The expression of HAMP is not static but is strictly regulated by the Bone Morphogenetic Protein (BMP)/Hemojuvelin (HJV)/Sma- and Mad-related proteins (SMAD) signaling pathway (26, 27). HJV is a common receptor of BMP; by enhancing the expression of BMP, it stimulates the SMAD signaling pathway and promotes the expression of HAMP (28). The process of BMP stimulating HAMP expression is also regulated by the hemochromatosis protein (HFE) (29). In an environment rich in iron, Tf competes with HFE for TFR1, resulting in the separation of HFE and TFR1 and the binding of TFR2, thus activating the SMAD signaling pathway and inducing the expression of HAMP (30). Membrane serine protease 2, also known as TMPRSS6, inhibits the expression of HAMP by lysing HJV and negative feedback (31). The abnormality of any gene in the HAMP regulatory signal transduction pathway will affect the expression of HAMP, eventually causing diseases related to iron metabolism disorders in the body. This precise molecular regulation mechanism ensures that cells can quickly make adaptive metabolic responses in the physiological states of iron-sufficient and iron-restricted.

### 2.3 Mechanisms of abnormal brain iron metabolism

#### 2.3.1 Oxidative stress and neuroinflammation

Iron in the human body exists mainly in the form of divalent iron or trivalent iron. Under pathological conditions, Fe^2+^ is easily oxidized by oxygen and H_2_O_2_ produced by cellular metabolism, forming Fe^3+^ and reactive oxygen species (ROS) such as superoxide anions and hydroxyl radicals through the Fenton reaction (32). The ROS induced by iron further promote oxidative stress, and this process will further promote the release of iron from ferritin and heme iron (33), thereby forming a toxic positive cycle between iron metabolism and oxidative stress, exacerbating cellular and tissue damage. This is the main mechanism by which abnormal iron metabolism leads to neurological damage (9). The imbalance of iron metabolism in the body, especially the increase of free Fe^2+^, can cause toxic effects on cells (34). At the same time, iron overload can activate microglia and astrocytes, releasing a large amount of pro-inflammatory factors such as TNF-α, IL-1β, and IL-6, exacerbating the neuroinflammatory response (35). This vicious cycle of oxidative stress and neuroinflammation ultimately leads to neuronal dysfunction and death (36).

#### 2.3.2 Mitochondrial dysfunction

Mitochondria are the energy production centers within cells, responsible for generating adenosine triphosphate (ATP) required by cells (37). Besides playing a central role in metabolism, mitochondria are also involved in other cellular processes such as Ca^2+^ homeostasis, redox signal transduction, and cell death, as well as influencing various iron-related functions (38, 39). Iron is a key cofactor in multiple complexes of the mitochondrial electron transport chain. Abnormal iron metabolism can disrupt mitochondrial function, leading to reduced ATP production and increased ROS generation (40). Excessive production of oxygen-free radicals can damage the structure and function of mitochondria, resulting in loss of mitochondrial membrane potential, disruption of the respiratory chain, reduced ATP production, and impairment of cellular function and energy crisis (41). In PD, iron overload is closely related to the inhibition of mitochondrial complex I function, which is the basis of the selective vulnerability of dopaminergic neurons (42). Moreover, a morphological and biochemical study of muscle biopsies from patients with depression showed that there were defects in brain energy metabolism, decreased activity of respiratory chain enzymes, reduced ATP production, and increased loss of mitochondrial DNA (43).

#### 2.3.3 Abnormal protein aggregation

In neurodegenerative diseases, there is a profound and complex interaction between iron and abnormal protein aggregation (44). Iron not only indirectly promotes abnormal protein aggregation through oxidative stress but also directly binds to key pathological proteins such as β-amyloid protein, tau, and α-synuclein, altering their conformation and accelerating their aggregation into toxic oligomers and fibrils (45). In AD, Fe^3+^ can coordinate and bind to specific amino acid residues on the Aβ peptide segment. This accelerates the transformation of Aβ from monomers to β-fold structures and fibrils (46). Moreover, experimental studies have shown (47) that Fe^3+^ can directly induce the aggregation of overphosphorylated tau protein. This combination of iron with Aβ and tau protein accelerates their aggregation and enhances their neurotoxicity (48). In PD, *in vitro* studies have demonstrated (49) that both divalent and trivalent iron ions significantly accelerate the fibrillation of α-synuclein. Iron interacts with α-synuclein, promoting the formation of Lewy bodies (50), which are hallmark features of synucleinopathies such as Parkinson’s disease, Lewy body dementia, and multiple system atrophy (51). Furthermore, in AD and PD patients, these abnormal protein aggregates further disrupt the cellular iron homeostasis, forming a positive feedback loop and accelerating the progression of the disease (52).

### 2.4 Toxicity mechanism of iron-dependent death

#### 2.4.1 Definition and core regulation of ferroptosis

Ferroptosis is a regulated form of cell death characterized by the accumulation of iron-dependent lipid peroxides (53). Its core biochemical feature involves the peroxidation of polyunsaturated fatty acids (PUFAs) on the cell membrane, catalyzed by iron ions. This leads to the substantial accumulation of toxic lipid peroxides, ultimately disrupting membrane structure and inducing cell death (54). This mode of death is fundamentally distinct from apoptosis, necroptosis, or autophagy in terms of its morphology and mechanism (55). The key regulatory node of it lies in the intracellular antioxidant defense system, especially glutathione peroxidase 4 (GPX4). As a selenium-dependent enzyme, GPX4 uses reduced glutathione (GSH) to reduce lipid peroxides into non-toxic lipids, thereby forming the main defense line against ferroptosis (56). When the function of GPX4 is impaired or GSH is depleted, lipid peroxidation will become uncontrolled. At this point, it will irreversibly trigger ferroptosis in the cells (57).

#### 2.4.2 Neuronal susceptibility to ferroptosis

A large number of studies show that neurons are particularly sensitive to this process, which is related to their high oxygen consumption rate, membrane structures rich in PUFA, and active iron metabolism requirements (58, 59). In neurodegenerative diseases, multiple pathological factors converge and jointly trigger the toxicity mechanism of ferroptosis. For example, the imbalance of iron homeostasis in the brain leads to excessive accumulation of free divalent iron, which is catalyzed by the Fenton reaction to generate ROS, directly exacerbating lipid peroxidation. At the same time, the expression or activity of GPX4 is reduced, and the function of the system xCT, which is responsible for cystine uptake to synthesize GSH, is inhibited, weakening the antioxidant capacity of the cells (60). The synergy of these mechanisms constitutes a common pathological model of “iron-oxidation-cell death,” which can explain the non-apoptotic neuronal loss observed in various neurodegenerative diseases such as Alzheimer’s disease and Parkinson’s disease.

#### 2.4.3 Differences in ferroptosis across various neurodegenerative diseases

However, the role of ferroptosis varies significantly across different neurodegenerative diseases, and this variation is closely associated with the characteristic pathological changes of each disease (61). For instance, in Alzheimer’s disease (62), ferroptosis has been associated with Aβ and tau protein pathology. Studies have shown that iron accumulation can induce lipid peroxidation and a decrease in GPX4 activity, thereby triggering the ferroptosis pathway; conversely, iron accumulation not only enhances the neurotoxicity of β-amyloid (Aβ) but also promotes the hyperphosphorylation of tau protein, creating a vicious cycle (63). In contrast, ferroptosis is associated with the selective vulnerability of dopaminergic neurons in the substantia nigra of individuals with Parkinson’s disease. Specifically, oligomerized α-synuclein promotes the production of reactive oxygen species and lipid peroxidation, thereby increasing neuronal susceptibility to ferroptosis (64, 65). Meanwhile, iron deposition primarily occurs in the substantia nigra pars compacta (66). Recent studies have further revealed that ferroptosis is not driven by a single factor but results from the synergistic action of multiple factors, including neuroinflammation, protein aggregation, mitochondrial dysfunction, and iron accumulation (67). This multifactorial process explains why, despite similar terminal pathways of ferroptosis across different diseases, their upstream triggering mechanisms and intervention targets exhibit significant differences (68).

## 3 Abnormalities in cerebral iron metabolism and neuropsychiatric disorders

### 3.1 Neurodegenerative diseases

#### 3.1.1 Parkinson’s disease

As a common neurodegenerative disease, Parkinson’s disease, its core pathological feature is the progressive loss of dopaminergic neurons in the substantia nigra compacta and the formation of Lewy bodies (69). A large number of neuropathological studies have confirmed that there is a significant iron deposition phenomenon in the brains of patients with Parkinson’s disease, especially in the substantia nigra region (70–72). Using high-field MRI technology and quantitative susceptibility mapping techniques from modern neuroimaging, postmortem brain tissue from PD patients was analyzed, confirming a positive correlation between iron deposition levels and disease severity (73, 74). However, since different neuroimaging techniques such as QSM and R2* have different sensitivities and specificities in detecting iron deposition, the comparison of research results should be done with caution. Studies have shown that in terms of quantifying brain iron deposition, QSM has higher accuracy and sensitivity than R2* mapping. For example, in a Parkinson’s disease model, QSM can significantly distinguish the iron content in the substantia nigra of the lesion group from that of the control group, while the R2* value did not show a significant difference (75). Another clinical study also pointed out that QSM is superior to R2* mapping in detecting iron deposition in the substantia nigra of Parkinson’s disease patients and its correlation with clinical severity (76). This phenomenon has also been observed in various PD animal models (77, 78).

Multiple studies have confirmed dysfunction in various iron metabolism-related proteins in the brains of patients with Parkinson’s disease. In the substantia nigra, TfR1 and DMT1 expression is upregulated, while FPN1 expression is downregulated. This pattern of increased uptake and reduced efflux directly leads to iron overload in dopaminergic neurons of the substantia nigra (79). Furthermore, iron homeostasis-related proteins such as hemopexin and Tf are significantly dysregulated in the substantia nigra of PD patients (80). In peripheral tissues, plasma ferritin concentrations in patients with early-stage PD are significantly correlated with the pro-inflammatory cytokine interleukin-6 and the iron metabolism regulator hepcidin, suggesting that peripheral iron homeostasis disruption also contributes to the progression of Parkinson’s disease (81). Dysfunction of these proteins involved in cellular iron metabolism leads to increased iron uptake and reduced iron efflux, ultimately resulting in intracellular iron overload (80, 82). Moreover, PD patients generally have a defective antioxidant system, such as reduced activity of the cysteine/glutamic acid reverse transporter and reduced GSH levels (83, 84). This can affect the activity of the key regulator of ferroptosis (85, 86), GPX4, resulting in a decline in the neurons’ defense function against oxidative stress and an increase in their susceptibility to ferroptosis. In addition, the abnormal expression of iron metabolism-related genes such as TfR1 and FPN1 may further aggravate the sensitivity of ferroptosis and abnormal iron deposition.

Therefore, maintaining the stability of intracellular iron metabolism and iron homeostasis, and controlling the level of lipid peroxidation within the physiological range, is an important research direction at present, and it may help to reduce the susceptibility to neuronal ferroptosis (87). Interventions targeting iron metabolism pathways and key regulatory factors for ferroptosis, such as GPX4 and Nrf2 pathways, hold significant potential for the prevention and treatment of PD.

#### 3.1.2 Alzheimer’s disease

Alzheimer’s disease is a neurodegenerative disorder characterized primarily by progressive cognitive and memory impairments. It is particularly prevalent among the elderly population (88). With the acceleration of the aging population, the incidence of AD is increasing year by year, and it has become an important fatal disease along with cardiovascular and cerebrovascular diseases and malignant tumors (89). The characteristic pathological changes of AD mainly include the deposition of extracellular Aβ in the brain, forming senile plaques and intracellular neurofibrillary tangles (NFTs), as well as progressive neuronal loss in brain regions such as the cortex and hippocampus (90), with damage to the cholinergic system being the most prominent; however, the neuronal loss associated with cognitive decline involves multiple neurotransmitter systems (91), including glutamatergic, GABAergic, dopaminergic, noradrenergic, and serotonergic systems (92). In fact, the pathogenesis of AD has not yet been fully elucidated (93).

Disruptions in iron metabolism in the brain are considered one of the key components of the pathological process of Alzheimer’s disease (94, 95). The study found that the iron content in the brain of AD patients was significantly increased, especially in specific areas such as the amygdala, hippocampus, and cortex (54). Moreover, the MRI diagnostic technique has revealed that during the early stage of AD, when Aβ accumulates, there is often an increase in iron concentration (96, 97). And the iron deposits observed in the cerebral cortex and hippocampus of AD patients are consistent with the distribution and location of Aβ plaques (94, 97). Research shows that (98) the iron in the brain increases with age. Therefore, when summarizing these studies, the confounding factor of age must be taken into account, and the brain iron accumulation during the normal aging process should be distinguished from the AD-specific iron metabolism disorders.

There is a complex interaction between iron metabolism abnormalities and the core pathological proteins of Alzheimer’s disease. As early as 1992, Connor et al.’s research (99) demonstrated that in the brain slices of AD patients, the distribution of senile plaques formed by Aβ and iron in surrounding cells increased significantly, suggesting that there was iron deposition in the brains of patients with AD and the iron homeostasis was disrupted. Studies using animal models found that increasing the iron content in the brain can exacerbate the aggregation of Aβ and increase the death of nerve cells in the affected brain region (97), while the use of iron-chelating agents to reduce the iron level in the brain can significantly improve the symptoms of AD (100). At the same time, iron overload also exacerbates the dysfunction of tau protein. The increase in iron leads to lipid peroxidation, which further drives the increase of oxidative stress in AD. This might promote the increase of abnormal phosphorylation of tau protein and the formation of tau fibril lesions (101, 102). Amyloid precursor protein (APP) is a transmembrane protein widely expressed in neurons. Research shows that the lack of tau protein will affect the post-translational transport process of APP, causing it to stay in the endoplasmic reticulum and be unable to transport to the surface of the cell membrane (103). It has been reported that APP exhibits ferric oxidase-like activity *in vitro* and under certain experimental conditions and may assist in cellular iron efflux by stabilizing FPN1 on the cell membrane (104). Therefore, tau protein deficiency may affect the release of intracellular iron by affecting APP, leading to an increase in intracellular iron and further aggravating neuronal damage.

Iron may directly participate in the pathological process of AD by promoting Aβ aggregation and tau protein phosphorylation but also aggravates neuroinflammation and neuronal damage through oxidative stress mechanisms (24). This results in a potential toxic positive feedback loop between iron metabolism abnormalities and Aβ and tau pathologies, accelerating the progression of AD (52).

### 3.2 Mental disorders

There are fundamental differences in the pathological nature between mental disorders and neurodegenerative diseases. The core characteristic of neurodegenerative diseases is the degeneration and death of neurons in specific brain regions, such as the progressive loss of dopaminergic neurons in the substantia nigra in Parkinson’s disease; in contrast, mental disorders are typically not accompanied by significant neuronal death but rather manifest as structural reorganization of neural circuits or abnormalities in functional connectivity. Consequently, brain iron metabolism plays distinct roles in these two categories of diseases. In neurodegenerative diseases, iron homeostasis imbalance is often directly involved in pathological processes such as oxidative stress, mitochondrial dysfunction, and ferroptosis, which subsequently lead to neuronal death. In contrast, in psychiatric disorders, disturbances in brain iron metabolism may affect neural circuits by influencing processes such as myelination, neurotransmitter synthesis, and release.

#### 3.2.1 Depression

Depression is a highly heterogeneous mood disorder characterized by significant and persistent low mood. In addition to mood disturbances, patients often experience multidimensional changes, including anhedonia, cognitive impairment, sleep and appetite disturbances, fatigue, and somatic symptoms (105). This clinical diversity suggests that the pathophysiological mechanisms underlying depression may involve abnormalities in multiple brain regions and various neurobiological pathways (106).

Recent studies have shown that abnormal brain iron metabolism may be associated with the onset of depression (107). In patients with depression, ferritin expression is downregulated in the prefrontal cortex, while the expression of DMT1 and FPN1 exhibits regional variations (108). In peripheral tissues, multiple meta-analyses have also confirmed that serum ferritin levels are significantly reduced in patients with depression (109). Human studies have provided important clues regarding the link between brain iron metabolism and depression. Data from an epidemiological survey conducted by Kim et al. (110) show that dietary iron intake is negatively correlated with the risk of depression. This indicates that adequate iron levels are crucial for maintaining normal brain function and emotional regulation. Hidese et al. (111) conducted a study and found that the prevalence of depression in patients with iron deficiency anemia was higher than that in the non-iron deficiency anemia group. It is speculated that iron deficiency anemia may trigger depression by affecting the synthesis of two classic monoamine neurotransmitters, dopamine and serotonin. In addition, the significance of brain iron homeostasis during the critical period of development is also worthy of attention. The research of Larsen et al. (112) reported that iron levels in the basal ganglia of adolescents gradually increase with age, continuing until around age 25, and that lower iron concentrations during adolescence are significantly associated with cognitive decline in late adolescence. Because cognitive impairment is one of the core symptoms of depression, this finding suggests that disruptions in brain iron homeostasis during adolescence may increase the risk of developing depression by affecting cognitive development.

Building on human studies, animal experiments have further revealed the potential mechanisms by which disturbances in brain iron metabolism contribute to the development of depression. Li et al.’s research (113) confirmed that a high-iron diet can cause iron overload in the brain tissue of mice and subsequently observed that the neuronal structure and function in the cerebral cortex and hippocampus were disrupted, and the neuronal marker proteins were downregulated. Mice with depressive-like behaviors often have brain neuronal cell damage (114). Mechanistically, excessive iron accumulation can lead to an increase in the unstable iron pool and ROS, thereby causing neuronal damage (115). On the other hand, the combination of iron deficiency and chronic unpredictable mild stress (CUMS) can induce depressive episodes in mice and exacerbate depressive symptoms. Together, these studies suggest that both iron overload and iron deficiency in the brain may be involved in the disease process and play an important role in the pathogenesis of depression (116).

Overall, the existing evidence on depression is mixed: some studies indicate increased iron levels in specific brain regions, while others suggest peripheral or central iron deficiency. This contradictory pattern may reflect the high heterogeneity of depression or the differential distribution of iron metabolism disorders across different brain regions.

#### 3.2.2 Schizophrenia

Schizophrenia is a severe chronic and disabling mental illness characterized primarily by disorganized thinking and impaired reasoning, abnormal perceptions, emotional disturbances, and significant impairment in social functioning and behavior (117). Similar to depression, the research results on brain iron metabolism in schizophrenia show complexity. Some studies suggest that patients may have abnormal levels of brain iron. A recent 2025 study (118) utilizing QSM and PET imaging techniques found that individuals with schizophrenia exhibited significantly reduced magnetic susceptibility values in the substantia nigra region of the brain compared to healthy controls. This suggests a reduction in the iron content bound to non-neural melanin in these areas. In recent years, the development of MRI technology has provided a powerful non-invasive method for the detection of brain iron levels. MRI studies show that the iron content of the basal ganglia and thalamus of patients with early schizophrenia is significantly reduced, and this decrease is associated with the severity of symptoms (119). Genetic research also found that the risk genes of schizophrenia overlap with the genes related to iron metabolism, suggesting that iron metabolism abnormalities may be an important biological basis for the disease (10). In addition, some studies have shown that the expression of myelin-related genes in patients with schizophrenia is abnormal, and iron is an essential element for the formation of myelin. This suggests that there may be more complex links between iron metabolism, myelination, and disease risk (120).

However, some studies have also reported iron overload in certain areas of the brain, indicating that iron metabolism disorders may have regional specificity in schizophrenia and heterogeneity among different studies in schizophrenia. At present, it is difficult to draw a unified conclusion (10). This inconsistency may stem from two potential causes. First, brain region specificity is a key factor. Contrary to the aforementioned iron reduction, a 2022 7T-MRI study revealed significantly elevated magnetization intensity in the bilateral putamen of chronic schizophrenia patients, suggesting increased iron content in this region. This alteration was accompanied by disrupted iron metabolism within the cortico-striatal pathway (121). Second, disease stage and duration may influence the pattern of iron metabolism alterations. Differences may exist between first-episode and chronic patients, and some studies suggest that iron metabolism abnormalities may not be a secondary effect of medication but rather an intrinsic feature of the disease itself (122).

These seemingly contradictory findings can be integrated at the mechanistic level. Recently, scholars have proposed that iron metabolism abnormalities in schizophrenia may exhibit “regional selectivity.” Iron deficiency observed in the cell bodies of dopaminergic neurons in regions such as the substantia nigra and ventral tegmental area may lead to hyperfunction of striatal dopamine by affecting tyrosine hydroxylase activity (118). Conversely, in regions like the prefrontal cortex, iron homeostasis disruption may increase free iron levels and exacerbate oxidative stress (123).

In summary, iron dyshomeostasis in schizophrenia exhibits significant regional specificity and inter-study heterogeneity. Future research should integrate high-resolution imaging, large-sample data, and molecular pathology approaches to elucidate the specific roles of iron metabolism across different brain regions and disease stages, as well as its association with clinical symptoms.

### 3.3 Neurodevelopmental disorders

Neurodevelopmental disorders occur during the critical period of brain development, and their abnormal iron metabolism is fundamentally different from that of neurodegenerative diseases and mental disorders (124). The latter primarily manifests as progressive iron deposition and overload in specific brain regions, while the former is more commonly associated with iron deficiency during early life (125). The key role of iron homeostasis during this period lies in ensuring normal myelination, neurotransmitter synthesis, and synaptic development (126). Therefore, iron deficiency at this stage is more frequently of concern.

#### 3.3.1 Twitching disorder

Tourette syndrome is the most representative neurodevelopmental disorder among tic disorders. Its core clinical feature is the persistent presence of multiple motor tics and at least one vocal tic. The etiology is complex and involves multiple factors, such as genetics, immunity, environment, and psychology. It usually manifests in childhood. In recent years, the neurodevelopmental hypothesis related to the dopaminergic system has provided an important theoretical entry point for studying the role of iron homeostasis imbalance in its pathophysiology (127). As a key trace element in the development of the nervous system, iron maintains a stable state that is crucial to brain function. In autopsy cases, reduced TfR1 expression levels in the striatal region suggest impaired iron uptake (128). Under the condition of iron deficiency, the process of iron entering iron enzymes and hemoglobin protein is restricted, which will also affect the maturity of oligodendrocytes and the synthesis of myelin, resulting in a decrease in myelin production (129, 130). Disorders in neurometabolism and myelin production are the potential pathological basis of various neuropsychiatric diseases (125). Research indicates that iron deficiency may be associated with tic disorders (125, 131). A large-scale domestic study showed that the average serum ferritin of children aged 5 to 12 years old with convulsion disorder was significantly lower than that of healthy control children, and the incidence of low serum ferritin in the convulsion disorder group was higher than that in the healthy control group (132). Another study also supported this result (133) and pointed out that the severity score of Tourette syndrome was negatively correlated with serum iron and ferritin levels, suggesting that iron content might be related to the severity of symptoms. In individuals with a twitching disorder, the abnormality of iron metabolism is not limited to the periphery but may also extend to the central nervous system. Kanaan et al. (128) employed quantitative magnetic susceptibility imaging technology to compare 28 patients with Tourette syndrome and 26 normal controls. They found that the brain iron content in regions such as the substantia nigra, subthalamic nucleus, striatum, dentate nucleus, and globus pallidus of the patients’ cohort was reduced, and the iron content in the striatum was related to the severity of Tourette syndrome. Gorman et al. (134) found that the level of peripheral serum ferritin in patients with Thomsen syndrome decreased, and at the same time, the volumes of brain regions such as the putamen, sensorimotor cortex, middle temporal cortex, and subcortical areas, which were rich in iron content, were reduced. This indicates that insufficient iron content in the brain may coexist with changes in brain structure.

#### 3.3.2 Attention deficit hyperactivity disorder

ADHD is one of the most common neurodevelopmental disorders in childhood (135). From a pathophysiological perspective, iron is an essential cofactor for the synthesis of dopamine and norepinephrine, and these two neurotransmitters play a central role in the pathogenesis of ADHD. Therefore, theoretical speculation suggests that iron deficiency in the brain region may affect the synthesis and metabolism of monoamine neurotransmitters, thereby leading to core symptoms of ADHD such as inattention, hyperactivity, and impulsivity (125). Multiple studies have found that the serum ferritin levels of children with ADHD are significantly lower than those of healthy children, suggesting that insufficient iron storage may be related to the onset of ADHD (136). As a post-processing technique, QSM provides a clear visualization of the distribution of human magnetic susceptibility, particularly in iron-rich deep gray matter structures such as the caudate nucleus, putamen, globus pallidus, substantia nigra, red nucleus, and dentate nucleus, where tissue contrast is significantly enhanced. A prospective QSM study in 2022 (137) showed that compared with healthy children, children with ADHD had widespread reductions in iron content in multiple brain regions, including the frontal lobe, globus pallidus, caudate nucleus, substantia nigra, putamen, and hippocampus. These brain regions are involved in key functions such as cognitive control, motor regulation, and attention. Additionally, this study further found that the volumes of the frontal lobe and hippocampus of children with ADHD were smaller than those of healthy children. This suggests that abnormal brain iron levels may coexist with structural developmental changes. An MRI study (138) found that the iron content in the striatum and thalamus of untreated ADHD children was lower. It is speculated that iron deficiency may limit the activity of iron-dependent tyrosine hydroxylase, thereby weakening dopaminergic signal transmission, which is also a core pathophysiological feature of ADHD. A study (139) showed that iron supplementation therapy has been shown in some studies to be able to improve the symptoms of children with ADHD, especially beneficial for cognitive functions and attention. This provides some supportive evidence for the association between the iron status in the brain and ADHD symptoms. However, more research is needed to confirm its efficacy and mechanism.

## 4 Treatment strategies

To reduce the risk of neurodegenerative diseases caused by iron metabolism disorders, many studies have attempted to mitigate the series of changes caused by iron aggregation in the brain through iron chelation. The treatment approach of iron chelation is to prevent further uptake of iron in the brain, thereby reducing the increased susceptibility to diseases due to iron accumulation. Blocking or down-regulating TfR or up-regulating the binding of the ferritin-iron transporter to the blood-brain barrier may also be one of the methods to limit unnecessary iron uptake by the brain.

However, the clinical significance of iron chelation therapy requires careful evaluation. Preclinical studies have provided preliminary evidence of the neuroprotective effects of iron chelation therapy. Deferoxamine (DFO) was the first iron chelator to be used clinically; it reduces intracellular pools of labile iron by chelating free iron ions and promoting their excretion via urine and bile. In Parkinson’s disease, studies have shown that iron chelators such as DFO can increase the activity of dopaminergic neurons and reduce oxidative damage, thereby improving the motor function of PD patients (140). Results from a study on deferiprone (DFP), another oral iron chelator, showed that DFP significantly reduced oxidative stress, cellular damage, and unstable iron levels in MPTP-induced Parkinson’s disease mouse models, improved motor function, and increased dopamine levels in the substantia nigra and striatum (141).

However, the results of these preclinical studies have faced challenges in clinical translation. In a randomized clinical trial, 12-month treatment with the iron chelator DFP significantly reduced iron accumulation in the substantia nigra of PD patients but did not result in clinically meaningful improvements in motor function (142). In Alzheimer’s disease, the situation is similar. A 2024 phase II clinical trial found that although the 12-month DFP treatment successfully reduced the iron content in the hippocampus, it accelerated the cognitive decline of the patients, suggesting that reducing brain iron in such patients may be harmful (143). Recently, a one-year clinical trial provided the first evidence of the iron-chelating potential in ALS (144). Results from patients treated with deferoxamine for one year showed good safety; compared with the first three months of the untreated period, the ALS Functional Rating Scale and BMI scores decreased significantly after three months of treatment, although the overall clinical benefit requires validation in larger-scale studies (145). These results collectively indicate that the evidence strength of the neuroprotective effect of iron chelation therapy in clinical settings is much lower than what was expected in preclinical studies. Its application should be strictly limited to specific populations and closely monitored.

Iron supplementation has been shown to effectively alleviate symptoms of iron deficiency-related conditions, including depression and ADHD, with the most pronounced effects observed in children with comorbid iron deficiency. A systematic review and meta-analysis published in 2025, incorporating 18 studies, demonstrated that iron supplementation significantly improved anxiety, fatigue, and physical health-related quality of life in non-anemic children, adolescents, and menstruating adults. It also enhanced cognitive intelligence and short-term memory performance (146). For instance, E. Konofal et al. (147) conducted a study on 23 children aged 5 to 8 with ADHD. They randomly assigned the children to receive oral ferrous sulfate or a placebo for 12 weeks. The results showed that the ADHD children treated with ferrous sulfate had significant improvement in symptoms, while those given the placebo showed no significant change. However, the aforementioned meta-analysis also emphasized that the supplementation effect disappeared after excluding participants with iron deficiency, suggesting that iron supplementation should be based on an accurate diagnosis of iron deficiency. In depression, a recent study (148) suggests that a high-iron diet may be involved in the pathogenesis through influencing synaptic plasticity. However, there is insufficient evidence to prove that this dietary intervention can be transformed into a clinically effective method.

In conclusion, the current treatment strategies for iron metabolism disorders are mostly derived from preclinical mechanism studies. However, the clinical translation effects are complex. The efficacy and safety of these treatments need to be rigorously verified in large-scale clinical trials in the future. If these strategies are applied indiscriminately, they may not bring benefits and even pose risks. The latest perspective suggests that future therapeutic development should move beyond the simplistic “iron overload” paradigm toward precision regulation—specifically, identifying specific subgroups that genuinely benefit from iron chelation or supplementation, determining optimal timing and dosage for interventions, and carefully balancing the effects of interventions on both systemic and cerebral iron homeostasis (149).

## 5 Therapeutic significance and future prospects

In conclusion, the imbalance of brain iron homeostasis has become a crucial link in understanding the pathophysiological mechanisms of neurological and psychiatric diseases, which also opens up a highly promising new direction for future treatment methods. The existing research results indicate that for neurodegenerative diseases such as Alzheimer’s disease and Parkinson’s disease caused by iron overload, iron chelators show strong potential in alleviating disease progression in preclinical and clinical studies (150); for neurodevelopmental disorders such as ADHD related to iron deficiency, iron supplementation may significantly improve the symptoms of patients (10). These findings can all support the idea that regulating the homeostasis of brain iron metabolism may become a new type of treatment method distinct from traditional approaches.

The in-depth study of cerebral iron homeostasis not only provides a new theoretical basis for the treatment of the disease after the onset of the disease but also suggests that ferroptosis can be prevented through nutritional intervention and the activation of the endogenous antioxidant system. Adequate intake of dietary selenium can maintain the activity of GPX4 and resist lipid peroxidation. A preclinical study in 2022 (151) showed that GPX4 expression was significantly reduced in a Parkinson’s disease model and that selenium supplementation or the use of ferroptosis inhibitors provided significant protection for dopaminergic neurons (152). This suggests that nutritional screening and selenium supplementation for high-risk groups may become a future prevention strategy. Another study (153) shows that activating the endogenous cellular antioxidant system is also a way to prevent ferroptosis. Nuclear factor E2-related factor 2 is a key transcription factor that regulates the expression of antioxidant genes (154). The study found that natural compounds such as radiculin or small molecule Nrf2 activators can upregulate the expression of these protective proteins, thus enhancing the cell’s resistance to ferroptosis. This suggests that the future development of drugs with both antioxidant and Nrf2 activation functions may provide more complete neurological protection (155).

However, there are still many difficulties in translating this scientific theory into practical clinical applications. Firstly, brain iron metabolism has high regional specificity and dynamics. The core challenge is how to achieve precise and safe targeted intervention. Secondly, the discovery of new mechanisms such as ferroptosis has deepened our understanding of the role of iron-dependent cell death in diseases, but it has also further demonstrated the complexity of artificial intervention. Simple iron supplementation or reduction may trigger unpredictable side effects. Moreover, the significant differences in individuals’ genetic background, disease stage, nutritional status, and lifestyle will lead to increasingly individualized treatment of diseases in the future. Diagnostic and therapeutic methods will be more in line with the actual conditions of individual patients.

In the future, research in this field can make breakthroughs in the following directions: Firstly, develop non-invasive, quantitative biomarkers and imaging technologies that reflect dynamic changes in brain iron levels to enable precise, real-time monitoring of disease progression and treatment efficacy. Secondly, it is necessary to deeply analyze the roles of key regulatory molecules of iron metabolism, such as hepcidin and TfR, in specific brain regions and cells, and attempt to discover more specific drug treatment targets. Thirdly, rigorous animal model experiments or clinical trials should be carried out to verify the effectiveness and safety of different intervention strategies for specific patient subgroups. Finally, it is also possible to study the interactions between brain iron metabolism regulation and other systems, such as the balance of trace elements, neuroinflammation, and gut microbiota, in order to construct a more comprehensive and interconnected disease model and bring innovative and revolutionary strategic progress to the prevention and treatment of neurological and mental diseases.

Although targeting brain iron metabolism has opened up unprecedented prospects for the treatment of neuropsychiatric diseases, this field is still in the early stage of translation. The key challenge currently faced lies in overcoming the regional specificity and dynamic complexity of brain iron metabolism to achieve precise intervention. Future research should focus on three core directions: First, visualizing dynamic changes in brain iron through imaging and biopsy techniques; second, classifying patients based on genetic and nutritional backgrounds to explore personalized treatment strategies; third, integrating iron metabolism with systems such as neuroinflammation, oxidative stress, and gut microbiota to construct multidimensional disease intervention networks. Through precise monitoring, individualized stratification, and multi-target integration, brain iron regulation will transition from a theoretical hypothesis to a viable clinical therapeutic strategy.

## 6 Conclusion

Current research indicates that abnormal brain iron metabolism is closely related to the pathogenesis of various neurological and psychiatric disorders, which provides a solid theoretical basis for the development of new treatment strategies in the future. In neurodegenerative diseases, iron overload is the main manifestation, which leads to neuronal damage or even death through mechanisms such as oxidative stress, neuroinflammation, and abnormal protein aggregation. In mental disorders, iron deficiency may be the main manifestation, and iron deficiency affects neurotransmitter synthesis and energy metabolism.

There are still some unknowns and challenges in the research on brain iron metabolism in neuropsychiatric diseases. Firstly, the regulatory mechanism of brain iron metabolism is not yet fully clear and definite; secondly, the detection techniques for brain iron dynamics still need to be developed and improved; for the treatment strategies targeting iron metabolism disorders, more methods need to be explored and clinical trials verified, etc. Future research directions can utilize multi-omics technologies to conduct more in-depth analysis of the molecular mechanisms of iron metabolism in neuropsychiatric diseases, and also apply different intervention measures at different stages of disease progression. In-depth research on brain iron metabolism not only helps to understand the pathophysiological mechanisms of neuropsychiatric diseases, provides valuable prevention and treatment strategies for clinical practice but also offers potential directions for the development of new diagnostic markers and therapeutic targets and has broad prospects for clinical translation.
